# Zebrafish as a Model System for the Study of Severe Ca_V_2.1 (α_1A_) Channelopathies

**DOI:** 10.3389/fnmol.2019.00329

**Published:** 2020-02-07

**Authors:** Sidharth Tyagi, Angeles B. Ribera, Roger A. Bannister

**Affiliations:** ^1^Medical Scientist Training Program, Yale University School of Medicine, New Haven, CT, United States; ^2^Department of Physiology and Biophysics, University of Colorado School of Medicine, Aurora, CO, United States; ^3^Department of Pathology, University of Maryland School of Medicine, Baltimore, MD, United States; ^4^Department of Biochemistry and Molecular Biology, University of Maryland School of Medicine, Baltimore, MD, United States

**Keywords:** Ca_V_2.1, α_1A_, P/Q-type, channelopathy, familial hemiplegic migraine type 1, episodic ataxia type 2, vertebrate models, zebrafish

## Abstract

The P/Q-type Ca_V_2.1 channel regulates neurotransmitter release at neuromuscular junctions (NMJ) and many central synapses. *CACNA1A* encodes the pore-containing α_1A_ subunit of Ca_V_2.1 channels. In humans, *de novo CACNA1A* mutations result in a wide spectrum of neurological, neuromuscular, and movement disorders, such as familial hemiplegic migraine type 1 (FHM1), episodic ataxia type 2 (EA2), as well as a more recently discovered class of more severe disorders, which are characterized by ataxia, hypotonia, cerebellar atrophy, and cognitive/developmental delay. Heterologous expression of Ca_V_2.1 channels has allowed for an understanding of the consequences of *CACNA1A* missense mutations on channel function. In contrast, a mechanistic understanding of how specific *CACNA1A* mutations lead *in vivo* to the resultant phenotypes is lacking. In this review, we present the zebrafish as a model to both study *in vivo* mechanisms of *CACNA1A* mutations that result in synaptic and behavioral defects and to screen for effective drug therapies to combat these and other Ca_V_2.1 channelopathies.

## Introduction

P/Q-type Ca_V_2.1 channels are the predominant voltage-gated Ca^2+^ channel isoform present at the neuromuscular junction (NMJ) and most central synapses. Since Ca^2+^ flux *via* these channels is critical for neurotransmitter release (Llinás et al., 1981; Turner et al., 1992; Uchitel et al., 1992; Dunlap et al., 1994, 1995; Ludwig et al., 1997), mutations in the Ca_V_2.1 α_1A_ subunit would be expected to impact synaptic efficacy. However, as discussed in sections “Ca_V_2.1 Channel Composition” to “The Expanding Spectrum OF Ca_V_2.1-α_1A_ Channelopathies” the direct consequences of mutations on channel function and the resultant neurologic phenotypes vary significantly. For example, two well-studied channelopathies—episodic ataxia type 2 (EA2) and familial hemiplegic migraine type 1 (FHM1)—arise from point mutations in the *CACNA1A* gene that encodes the α_1A_ subunit (Jen et al., 2007; Pietrobon, 2007, 2010). The mutations that lead to EA2 tend to be loss-of-function mutations, while gain-of-function mutations usually underlie FHM1 (Jen et al., 2001; Tottene et al., 2002; Kaja et al., 2005, 2010; Mantuano et al., 2010; Rajakulendran et al., 2010b; Di Guilmi et al., 2014; Rose et al., 2014; Brusich et al., 2018). However, some ataxic cases have paradoxically been linked to gain-of-channel function mutations (e.g., van den Maagdenberg et al., 2010; Knierim et al., 2011; Gao et al., 2012; Bahamonde et al., 2015; Jiang et al., 2019). These latter examples underscore the diversity of channel dysfunction in this expanding spectrum of ataxic disorders and highlight the need for a model system to rapidly and effectively identify pathological phenotypes.

In this article, we review the: (1) basic information about the Ca_V_2.1 channel heteromultimer; (2) two relatively well-characterized diseases caused by mutation of the Ca_V_2.1 α_1A_ subunit—EA2 and FHM1; (3) the emerging full spectrum of Ca_V_2.1 α_1A_ channelopathies; and (4) the potential that the zebrafish model holds for understanding disease mechanisms and discovering potential therapeutics. Sections “Introduction” to “Familial Hemiplegic Migraine Type 1” are intended to provide sufficient background for the more profound discussion of the more severe neurodevelopmental disorders, which are caused by point mutations in *CACNA1A* in section “The Expanding Spectrum OF Ca_V_2.1-α_1A_ Channelopathies.” It is important to note that the pathology of this unnamed class of disorders resembles that of spinocerebellar ataxia type 6 (SCA), which is caused by the addition of excess CAG polynucleotide repeats to the *CACNA1A* transcript (Jodice et al., 1997).

## Ca_V_2.1 Channel Composition

**Figure 1:**
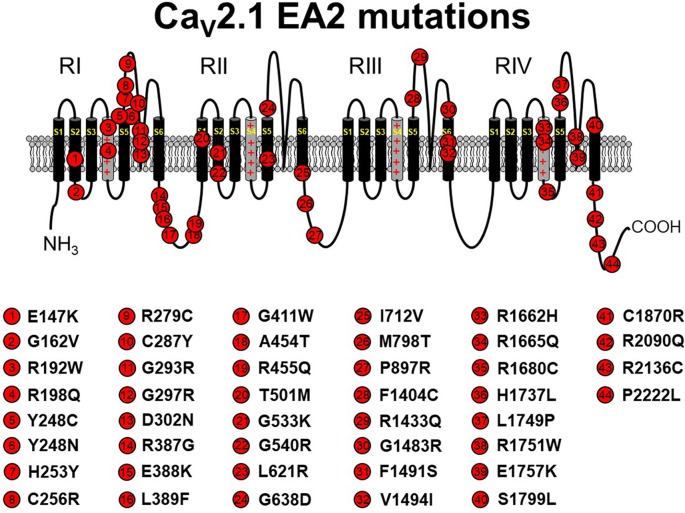
Schematic representation of human Ca_V_2.1 mutations causing episodic ataxia type 2 (EA2). Please note that residue numbering varies between studies due to the existence of multiple *CACNA1A* splice variants; residue numbers indicated reflect those stated in the original report. Citations to the indicated mutations are listed as follows: E147K—Imbrici et al., 2004; G162V—Maksemous et al. (2016); R192W—Soden et al. (2014); R198Q—Indelicato et al. (2019); Y248C—Zafeiriou et al. (2009); Y248N—Choi et al. (2017); H253Y—van den Maagdenberg et al. (2002); C256R—Mantuano et al. (2004); R279C—Maksemous et al. (2016); C287Y—Jen et al. (2004); G293R—Yue et al. (1997); G297R—Tantsis et al. (2016); D302N—Maksemous et al. (2016); R387G—Maksemous et al. (2016); E388K—Nikaido et al. (2011); L389F—Mantuano et al. (2010); G411W—Maksemous et al. (2016); A454T—Cricchi et al. (2007); R455Q—Isaacs et al. (2017); T501M—Mantuano et al. (2010); G533K—Scoggan et al. (2006); G540R—Rajakulendran et al. (2010a); L621R—Rajakulendran et al. (2010a); G638D—Cuenca-León et al. (2009); I712V—Guerin et al. (2008); M798T—Mantuano et al. (2010); P897R—Mantuano et al. (2010); F1404C—Jen et al. (2001); R1433Q—Pietrobon (2010); G1483R—Mantuano et al. (2004); F1491S—Guida et al. (2001); V1494I—Mantuano et al. (2004); R1662H—Friend et al. (1999); R1665Q—Tonelli et al. (2006); R1680C—Mantuano et al. (2010); H1737L—Spacey et al. (2004); L1749P—Maksemous et al. (2016); R1751W—Bertholon et al. (2009); E1757K—Denier et al. (2001); S1799L—Ohba et al. (2013); C1870R—Mantuano et al. (2010); R2090Q—Melzer et al. (2010); R2136C—Mantuano et al. (2004); P2222L—Sintas et al. (2017). The Ca_V_2.1 schematic was modified from Tyagi et al. (2019) with permission of the authors.

High voltage-activated Ca^2+^ channels, such as the Ca_V_2.1 heteromultimer, are composed minimally of a principal α_1_ subunit (α_1A_) and auxiliary β and α_2_δ subunits (Volsen et al., 1997; Catterall, 2010; Dolphin, 2016). For Ca_V_2.1, an interaction with a γ_2_ subunit (a.k.a., stargazin) was also reported (Letts et al., 1998; Kang and Campbell, 2003). Like the other nine members of the Ca_V_ family, α_1A_ subunits have four transmembrane repeats (I–IV), each with six membrane-spanning α-helices (S1–S6; Mori et al., 1991; please see Figure 1). Of these, the S4 α-helices are thought to be the primary voltage-sensing elements of the channel, a function which is conferred by five to six positively charged amino acids lining a face of the α-helix (Aggarwal and MacKinnon, 1996). The S1–S3 helices form an aqueous conduit that enables passage of the S4 α-helix through the membrane field by facilitating interactions with residues of the “charge transfer center” (formed by conserved negative, polar and hydrophobic residues on the S2 segment and an invariant aspartate residue on the S3 helix; Tao et al., 2010); the S5 and S6 helices line the conventional channel conduction pore (Neely and Hidalgo, 2014; Hering et al., 2018). The relatively long extracellular segment linking the S5 and S6 helices (a.k.a., the P-loop) contains a highly conserved glutamate residue in all four repeats. These four glutamates form the selectivity filter (Yang et al., 1993).

## Episodic Ataxia Type 2

EA2 is a rare neurological disease characterized by paroxysmal attacks of ataxia, nystagmus, and vertigo. The majority of *CACNA1A* mutations that lead to EA2 result in Ca_V_2.1 loss of function by premature termination of the open reading frame, resulting in rapid degradation of truncated protein products (Jen et al., 2001; Pietrobon, 2010; Sintas et al., 2017). Indeed, over 40 pathogenic missense mutations were identified (Pietrobon, 2010; Sintas et al., 2017; see Figure 1). Most of these amino acid substitutions reside in the P-loop or the S5 and S6 helices, themselves, suggesting that impaired ability to form a fully functional channel pore is the likely pathophysiological mechanism of the resultant phenotype for the majority of EA2 missense cases (Jen et al., 2007; Sintas et al., 2017). In some cases, a complete loss of function was observed with missense mutants, likely attributable to ER-associated degradation of the mutant channel and subsequent lack of trafficking to the surface membrane (Page et al., 2004). In addition, some EA2 mutants (e.g., E1761K, F1406C) seem to exert a dominant-negative effect since coexpression of mutant channels with wild-type channels in *Xenopus* oocytes diminished the amplitude of Ca^2+^ current elicited by depolarization (Jeng et al., 2006, 2008; Mezghrani et al., 2008). In these latter cases, it was postulated that misfolded mutant channels bound wild-type channels and subsequently induced degradation (Page et al., 2010; Rajakulendran et al., 2012; Dahimene et al., 2016) or competed successfully with the wild-type channel for a limited number of “slots” reserved for Ca_V_2.1 channels at the plasma membrane (Cao et al., 2004; Cao and Tsien, 2010; but see below). In addition, some mutations (e.g., H1736L, A1293D/delY1294, G293R) do not completely abolish channel activity but rather shift the voltage-dependence of Ca_V_2.1 activation to somewhat more positive potentials, thereby decreasing channel open probability (P_o_; Wappl et al., 2002; Spacey et al., 2004; Pietrobon, 2010).

In a minority of cases, EA2 is precipitated by gain-of-channel function mutations, which suggests that a critical bandwith of Ca^2+^ flux is required to avoid pathogenicity (e.g., Mantuano et al., 2010; Knierim et al., 2011; Gao et al., 2012; Carreño et al., 2013; Bahamonde et al., 2015). For many yet-to-be characterized Cav2.1 EA2 mutations, whether the mutation produces gain- or loss-of-channel function remains to be seen. Still, these findings underscore the need to resist generalization regarding pathological mechanisms without rigorous investigation of each mutation.

## Familial Hemiplegic Migraine Type 1

**Figure 2:**
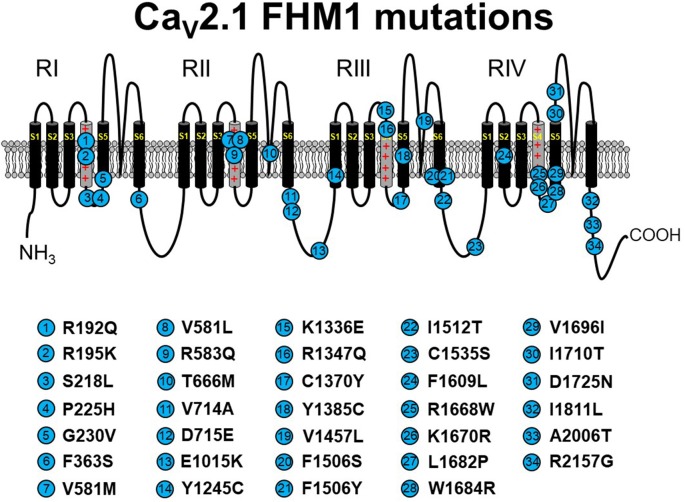
Schematic representation of human Ca_V_2.1 mutations causing familial hemiplegic migraine type 1 (FHM1). Please note that residue numbering varies between studies due to the existence of multiple *CACNA1A* splice variants; residue numbers indicated reflect those stated in the original report. Citations to the indicated mutations are listed as follows: R192Q—Ophoff et al. (1996); R195K—Ducros et al. (2001); S218L—Kors et al. (2001); P225H—Stuart et al. (2012); G230V—Yang et al. (2014); F363S—Riant et al. (2010); V581M—Cuenca-León et al. (2008); V581L—Freilinger et al. (2011); R583Q—Battistini et al. (1999); T666M—Ophoff et al. (1996); V714A—Ophoff et al. (1996); D715E—Ducros et al. (2001); E1015K—Grieco et al. (2018); Y1245C—Cuenca-León et al. (2008); K1336E—Ducros et al. (2001); R1347Q—Alonso et al. (2004); C1370Y—Thomsen et al. (2007); Y1385C—Vahedi et al. (2000); V1457L—Carrera et al. (1999); F1506S—Riant et al. (2010); F1506Y—Pelzer et al. (2018); I1512T—Grieco et al. (2018); C1535S—Dichgans et al. (2005); F1609L—Pelzer et al. (2018); R1668W—Ducros et al. (2001); K1670R—Riant et al. (2010); L1682P—Weiss et al. (2007); W1684R—Ducros et al. (2001); V1696I—Ducros et al. (2001); I1710T—Kors et al. (2004); D1725N—Riant et al. (2010); I1811L—Ophoff et al. (1996); A2006T—Wilson (2014); R2157G—Grieco et al. (2018). The Ca_V_2.1 schematic was modified from Tyagi et al. (2019) with permission of the authors.

FHM1 is an inherited migraine condition that results in weakness of half the body for prolonged periods of time. Patients afflicted with FHM1 often display cerebellar degeneration (Elliot et al., 1996). As noted above, FHM1 is most often linked to gain-of-function point mutations in *CACNA1A* (Tottene et al., 2002; Pietrobon, 2007; see Figure 2). These substitutions occur at a variety of loci within the channel but most commonly in residues thought to line the pore, the S3–S4 or S5–S6 linkers, or the S4 voltage sensor. Even though the locations of the mutations within the channel are variable, analysis in heterologous systems revealed a hyperpolarizing shift in channel activation for most studied mutants (Hans et al., 1999; Tottene et al., 2002, 2005; Adams et al., 2009; Serra et al., 2009). Since these channels open at more hyperpolarizing potentials, channel *P*_o_ is enhanced, and an FHM1 mutant Ca_V_2.1 channel can carry greater Ca^2+^ influx than its wild-type counterpart at physiologically relevant membrane potentials. This process may be further facilitated by a reduction in the direct Gβγ-mediated inhibition of presynaptic FHM1 mutant Ca_V_2.1 channels (Melliti et al., 2003; Weiss et al., 2008; Serra et al., 2009; Garza-López et al., 2012, 2013). Mouse knock-in models carrying FHM1-causing Ca_V_2.1 mutations display the migraine aura, cortical spreading depression characteristic of human FHM1 (van den Maagdenberg et al., 2004, 2010). While these gain-of-function biophysical effects of FHM1 mutations are fairly consistent, it is important to state that FHM1 pathology is inarguably a reflection of the balance of the relative manifestation of the mutations between excitatory and inhibitory circuits (Vecchia et al., 2015).

## The Expanding Spectrum of Ca_V_2.1-α_1A_ Channelopathies

**Figure 3:**
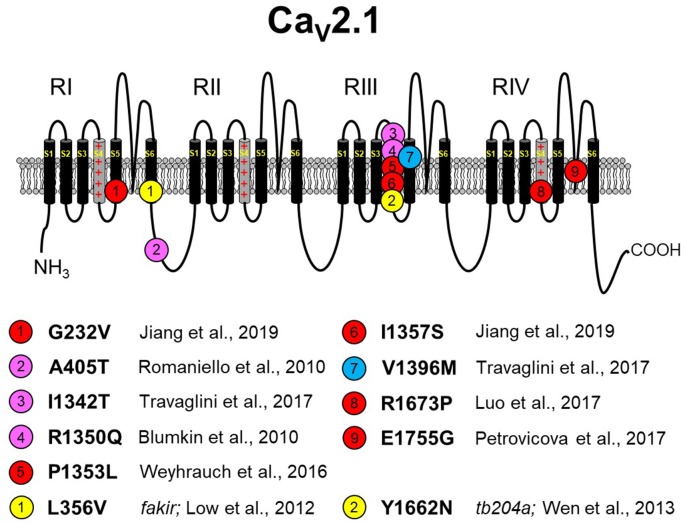
Missense Ca_V_2.1 mutations leading to neurodevelopmental disorders. The zebrafish *fakir* and *tb204a* mutants are also depicted as yellow circles. Red circles indicate a loss-of-function human mutation. Blue circles indicate a gain-of-function human mutation. Magenta circles indicate a yet-to-be functionally characterized human mutation. Specific references are indicated below. As in Figures 1, 2, please note that residue numbering varies between studies due to: (1) the existence of multiple known *CACNA1A* splice variants; and (2) species differences between humans and zebrafish. The Ca_V_2.1 schematic was modified from Tyagi et al. (2019) with permission of the authors. These mutations are discussed in sections “The Expanding Spectrum OF Ca_V_2.1-α_1A_ Channelopathies” and “Zebrafish as a Model SYSTEM for the Study of Severe Ca_V_2.1 Channelopathies.”

EA2 and FHM1 have long been known to be caused primarily by point mutations in Ca_V_2.1 in addition to a few variants that carry deletions or insertions (Jen et al., 2001; Pietrobon, 2007, 2010). However, the biophysical effects of these mutations on channel function are often subtle, and the manifestations of ataxia are paroxysmal (Elliot et al., 1996; Jen et al., 2007; Sintas et al., 2017). With the innovative whole-exome sequencing approach, a new, but yet-to-be-named, class of Ca_V_2.1-linked disorders with developmental components was identified and linked to point mutations in Ca_V_2.1 (Tonelli et al., 2006; Blumkin et al., 2010; Romaniello et al., 2010; Epi4K Consortium and Epilepsy Phenome/Genome Project, 2013; Damaj et al., 2015; Jiang et al., 2016; Weyhrauch et al., 2016; Luo et al., 2017; Travaglini et al., 2017). These disorders represent the far end of the Ca_V_2.1 channelopathy spectrum, which includes FHM1 and EA2. As is the case with spectrum disorders, these more severe disorders often share the characteristics of migraine and ataxia with FHM1 and EA2, respectively. However, the more severe disorders display cognitive deficits, epilepsies, and neurodegeneration that are infrequently observed with FHM1 and EA2 patients. Though similar in presentation, disorders resulting from Ca_V_2.1 missense mutations differ in etiology from SCA6, which is caused by increasing polyglutamine expansions on the channel carboxyl-terminus (Jodice et al., 1997; Frontali, 2001). Moreover, the scattering of mutations within the channel suggests that there are a variety of mechanisms for channel dysfunction underlying this class of disorders (Figure 3). For example, Romaniello et al. (2010) described an A405T substitution in a 12-year-old girl with a family history of Ca_V_2.1 mutation-linked disorders. The patient presented with persistent cerebellar signs (i.e., ataxia, dysmetria, hypotonia) and developmental delay. A405T represents a non-polar to polar substitution in the Repeat I–II linker region of Ca_V_2.1 (Figure 1). The Repeat I–II linker is putatively the site where the auxiliary β subunit interacts with the α_1A_ subunit (Campiglio and Flucher, 2015). A reasonable, but yet-to-be-tested, hypothesis is that the A405T substitution disrupts the α_1A_-β subunit interaction in much the same way as does an engineered Y392S swap in the I–II loop (Pragnell et al., 1994). Such a disruption would substantially decrease surface expression of the channel by impeding trafficking and, given reduced production of the wild-type protein, would likely result in haploinsufficiency. An alternate explanation is that the A405T substitution that impacts neurotransmitter release, similar to another ataxic variant in the I–II linker, A454T, was demonstrated to curb modulation of Ca_V_2.1 by SNARE proteins *via* a mechanism involving the β subunit (Cricchi et al., 2007; Serra et al., 2010, 2018).

Blumkin et al. (2010) reported a R1350Q substitution in a 7-year-old male patient that also presented with cerebellar ataxia, developmental delay, and nonspecific dyskinesia. Although the outward presentation was similar to the patient carrying the A405T substitution, the R1350Q swap inserted a neutral glutamine in place of a basic arginine in the S4 voltage-sensing α-helix of Repeat III (Figure 1). An arginine to glutamine substitution at this position was also reported with a patient exhibiting tremor that was alleviated by a Ca^2+^ channel blocker (R1345Q in Jiang et al., 2016). Based on the observation that the equivalent substitution in the *tottering* mutant mouse causes a ~12-mV hyperpolarizing shift in activation (Miki et al., 2008), it is likely that neutralization of this basic residue may have facilitated the movement of the voltage sensor through the membrane field. Such gain of function contrasts with the findings of Weyhrauch et al. (2016), who also described a mutation in the S4 voltage sensor of Repeat III (P1353L) found in a child with developmental delay, gross motor delay, and congenital hypotonia (Figure 1). Electrophysiological analysis of mutant channels expressed heterologously in HEK293 cells revealed near 100% ablation of Ca_V_2.1-mediated Ca^2+^ current, suggesting that either dominant-negative effects or haploinsufficiency underlies the phenotype. The first possibility was proposed on the basis that mice with only one *CACNA1A* allele seems normal (Jun et al., 1999). However, the ability of Ca_V_2.1P1353L to out-compete endogenously wild-type channels was not investigated in a neuronal context.

Travaglini et al. (2017) reported a pair of mutations, I1342T and V1396M, in two patients with similar clinical phenotypes involving congenital ataxia, hypotonia, and intellectual disability. The I1342T mutation resides in the extracellular loop between the S3 and S4 helix of α_1A_ in close proximity to the beginning of the Repeat III S4 helix (Figure 1). A reasonable hypothesis for the dysfunction of the I1342T mutant channel is that this substitution alters the conformation of the S4 helix and affects its mobility, though speculation on its relationship to ataxia, hypotonia, and intellectual disability is unfounded without more biophysical information regarding mutant channel dysfunction. The V1396M mutation is found in the proximal S5 pore-forming domain of Repeat III of α_1A_, a region of the channel that is also predicted to interact with the α_2_δ subunit on the basis of Ca_V_1.1 cryo-EM structure (Wu et al., 2016). The idea that V1396M facilitates channel expression through an α_2_δ-mediated mechanism (see Dolphin, 2016, for a review) is particularly intriguing since the current density for the mouse equivalent of Cav2.1 V1396M expressed in HEK293 cells was shown to be nearly double that of wild-type Cav2.1 (Jiang et al., 2019). Though less striking, the introduction of methionine also causes a hyperpolarization in the voltage dependence of activation suggesting the disruption of an inter-helical interaction that restricts voltage-sensor translocation. Three other Ca_V_2.1mutants, which were linked to Lennox–Gastaut epileptic encephalopathy were examined in the same study and were found to have polar effects (Jiang et al., 2019). The A715T mutation at the base of RIIS6 displayed a ~10-mV hyperpolarizing shift in activation, smaller but reminiscent of the ~20-mV hyperpolarizing shift observed in Purkinje cells of Ca_V_2.1 S218L EA2 model mice (Gao et al., 2012). On the other hand, G232V and I1357S, at the bases of RIS5 and RIIS4 helices, respectively, reduced channel plasma membrane expression in both HEK293 and in cortical neurons.

Seminal work from Richard Tsien’s laboratory in the early 1990s revealed that four highly conserved glutamate residues within the P-loop are the structural basis of Ca^2+^ selectivity among all Ca_V_ channels (Yang et al., 1993). Two such mutations in α_1A_ are known to occur at the same glutamate in Repeat IV. Mutation of this residue to glycine causes ataxia and cognitive deficits running through three generations of the Slovak family (E1755G in Petrovicova et al., 2017), and as noted above, a reversal of charge *via* substitution of a lysine for the glutamate causes EA2 (E1761K in Denier et al., 2001). The glutamate to lysine mutation ablates inward Ba^2+^ flux *via* the channel in *Xenopus* oocytes (Jeng et al., 2006). Since coexpression of the Ca_V_2.1 E1761K mutant with the wild-type channel reduced the amplitude of the current in an RNA dose-dependent manner, the authors postulated that the E1761K resulted in a dominant-negative effect. While this mechanism could certainly underlie this particular channelopathy, conversion of any one of the glutamates in the selectivity filter to lysine effectively transforms Ca_V_ channels into non-specific monovalent ion channels that are subject to block by divalent ions (Yang et al., 1993). In this regard, Jeng et al. (2006) used a concentration of Ba^2+^ (40 mM) in their experiments showing the ablation of inward current *via* E1761K channels, which most likely would have blocked the mutant channel. At more physiological divalent ion concentrations (i.e., <2 mM Ca^2+^), currents carried by Na^+^ and K^+^ might be visible and pathogenic. Indeed, aberrant Na^+^ and K^+^ flux *via* Ca_V_1.2 Repeat III glutamate to lysine mutant channels can prolong action potential duration in cardiac-like iPSCs (Ye et al., 2019), while the equivalent mutation in Ca_V_1.1 is postulated to cause K^+^ accumulation in the transverse tubules (Beqollari et al., 2018) and to accelerate muscle fatigue in mice (Lee et al., 2015). Thus, the possibility that the E1761K mutation augments neurotransmitter release by prolonging neuronal action potential duration is not unreasonable, nor is the idea that excessive K^+^ secretion into restricted extracellular compartments may excite neighboring neurons or vascular smooth muscle cells (see Filosa et al., 2006).

Recently, Luo et al. (2017) described an 8-year-old female patient with congenital ataxia, hypotonia, cerebellar atrophy, and global developmental delay. The trio-based exome sequencing of this patient revealed a *de novo* missense mutation (R1673P) in the gene for Ca_V_2.1. The mutation resulted in an arginine to proline substitution within the Repeat IV S4 voltage-sensing helix of Ca_V_2.1. The R1673P mutation was predicted to be “probably damaging” by PolyPhen-2, a protein structure prediction software. As a means to identify the molecular mechanism by which R1673P precipitates the clinical phenotype, transgenic flies expressing the *Drosophila* equivalent of wild-type Ca_V_2.1 and Ca_V_2.1 R1673P in a Ca_V_2.1-deficient *Drosophila* (i.e., *cacophony* mutants) background were generated. In these experiments, the mutant Ca_V_2.1 R1673P was able to rescue the photoreceptor response in 3-day-old larvae to a greater extent than the wild-type channel suggesting a gain-of-function effect. At 30 days, the rescue of the electroretinogram had dissipated, but substantial photoreceptor degeneration was observed in the R1673P line but not in wild-type or Ca_V_2.1-deficient flies. It is possible that the early effects of gain-of-function Ca^2+^channel activity triggered neurodegeneration secondary to Ca^2+^ toxicity. In contrast, however, voltage-clamp experiments showed that the R1673P mutation causes a profound loss-of-function for channels expressed heterologously in tsA-201 cells (Tyagi et al., 2019). Specifically, the rat ortholog of R1673P (R1624P) displayed a ~25-mV depolarizing shift in activation and resultant weak activation at physiologically relevant membrane potentials. Further work is needed to understand how the loss of function at the molecular level leads to neurodegeneration at the systemic level.

## Zebrafish as a Model System for the Study of Severe Ca_V_2.1 Channelopathies

Heterologous expression systems are the industry standard for the identification of pathogenic channel dysfunction. However, it is often difficult to extrapolate information gleaned using this approach to neurological dysfunction in patients. To bridge this gap, animal models are employed. Mice carrying FHM1 or EA2 mutations were very useful in understanding the pathophysiology underlying these disorders. However, no mouse line yet exists that models the more severe developmental disorders discussed above. The paucity of such models may be due to the uncertain viability or breeding capability of mice with grave developmental defects and the monetary risk associated with this endeavor. By contrast, simpler organisms like *Drosophila* have rapid propagation, are relatively easy to manipulate genetically, and lack the burden of cost. The obvious shortcoming of *Drosophila* is that insects are both phylogenetically and physiologically far removed from humans. A notable shortcoming is that *Drosophila* lack a true Ca_V_2.1 channel (Smith et al., 1996).

Zebrafish—*Danio rerio*—offers a unique complement to the strengths of flies and mice as models for the study of severe Ca_V_2.1 channelopathies. The zebrafish is useful to investigate mechanisms because of the conservation of most fundamental physiology processes (e.g., neurotransmitter release) with mammals with a reduced risk of embryonic lethality. Similar to many zebrafish genes, the gene encoding the Ca_V_2.1 α-subunit is duplicated, yielding *cacna1aa* and *cacna1ab*. Two zebrafish loss-of-function *cacna1ab* mutants, *tb204a* (Wen et al., 2013) and *fakir* (Low et al., 2012), were studied previously. For both mutations, the loss-of-channel function was sizable, but incomplete. The *tb204a* mutation results in a tyrosine-to-asparagine substitution (Y1662N) within the carboxyl terminus of Ca_V_2.1a and a depolarizing shift in channel activation, similar to what was found for the rat cognate of Ca_V_2.1 R1673P (Tyagi et al., 2019). Homozygous *cacna1ab^tb204a−/−^* larvae were viable and had reduced motility. Moreover, there was an increased incidence of synaptic failure at the NMJ due to reduced Ca^2+^ flux into the presynaptic NMJ, as detected by imaging of presynaptic intracellular Ca^2+^ (Wen et al., 2013). While this defect accurately predicted reduced motor function, neither sensory nor central effects of the mutation were assessed so their potential contribution to the behavioral phenotype cannot be excluded. Interestingly, both swimming behavior and NMJ synaptic transmission were rescued in *cacna1ab^tb204a−/−^* larvae by 3,4-diaminopyridine (a K^+^ channel blocker) and Roscovitine (a P/Q-type channel agonist; Yan et al., 2002; Buraei et al., 2007; Tarr et al., 2013).

The *fakir cacna1ab* mutation results in a L356V substitution in the S6 helix of Repeat I (Figure 1). Like the *tb204a* larvae, *fakir* mutants display reduced locomotor behavior compared to wild-type siblings. In addition, heterologously expressed *fakir* and *tb204* mutant channels had reductions in current amplitude and similar depolarizing shifts in channel activation properties (Low et al., 2012; Wen et al., 2013). *a priori*, L356V would appear to be a conservative amino acid change. However, L356 (located at the cytoplasmic side of S6 in RI) is highly conserved across species. Interestingly, the *tb204a* mutation (Y1662N) resides in an analogous location in S6 of RIV. While no disease-causing mutations have yet been identified in RIS6, human pathogenic point mutations were detected in the S6 helices of Repeats II–IV (Figures 1–3). Two of the mutations in S6 domains, V1494I and I1811L, would, similar to *fakir*, also be considered to be conservative substitutions. Overall, despite the identification of several S6 mutations, how L356V or other S6 mutations lead to perturbed channel function remains unknown. However, the fact that this is a highly conserved region across species suggests that mutations, even conservative ones, would be of consequence.

Despite the somewhat similar effects on channel activity produced by the two different *cacna1ab* mutations, substantially different mechanisms were proposed for how channel dysfunction leads to abnormal locomotor behavior. Consistent with the behavioral immotility, Low et al. (2012) found that rigorous swimming could be evoked in wild-type, but not *fakir* mutant, slow-twitch muscle by tactile stimulation. However, examination of responses to direct application of acetylcholine as well as miniature end plate current properties revealed little differences in transmission between motor neurons and slow-twitch fibers in *fakir* vs. wild-type larvae, nor were defects detected in evoked transmission between CaP motor neuron and fast-twitch muscle fibers. On this basis and consistent with the initial identification of *fakir* as a reduced touch-sensitive mutant (Granato et al., 1996), Low et al. (2012) proposed that *fakir* mutants have defective swimming responses to tactile stimulation because the relevant sensory neuron Rohon–Beard cell required *cacna1ab* for function. However, this hypothesis was not tested directly by recording from Rohon–Beard neurons or their post-synaptic partners. In contrast, a study of the *tb204* allele provided strong evidence to support defective transmission at the NMJ (Wen et al., 2013). Supporting evidence was provided by paired recordings between one type of motor neuron, CaP, and its fast-muscle target cell. Whether similar transmission defects occur at the NMJs formed between other motor neurons and muscle targets has not been studied. Thus, the mechanistic bases for the reduced motility defects of *fakir* and *tb204a* mutants have not been resolved.

Despite this impasse, the viability of both the *fakir* and the *tb204* mutant lines bodes well for the potential usefulness of zebrafish larvae carrying missense mutations corresponding to those which cause severe human Ca_V_2.1 channelopathies (e.g., Ca_V_2.1 R1673P). The generation of such models through CRISPR-Cas9 technology would enable the study of individual mutations with approaches encompassing the molecular, systemic, and behavioral levels. In particular, *via* paired CaP motor neuron—muscle recordings and imaging of depolarization-induced Ca^2+^ flux into presynaptic terminals allow assessment of whether impairments in locomotor function result from NMJ defects.

Since zebrafish were successfully used to screen for compounds for the treatment of Dravet syndrome, a *SCNA1A*Na^+^ channelopathy (Griffin et al., 2017), one can envision that this approach could be used to identify and/or refine small molecules to combat both Ca_V_2.1 gain- and loss-of-function disorders. Compounds that partially counteract channel gain of function, notably gabapentin and pregabalin, were available for clinical use for sometime (Sills, 2006). However, a need for alternatives arose as both the aforementioned compounds were shown to have some addictive capability (Bonnet et al., 2018; Althobaiti et al., 2019). In regard to loss-of-function disorders, 3,4-diaminopyridine was approved for acute treatment of Lambert—Eaton syndrome, a condition secondary to an aggressive lung cancer in which autoantibodies to Ca_V_2.1 are generated (García and Beam, 1996; Maddison, 2012). Unfortunately, the arrhythmogenic potential of this compound precludes its long-term use in other contexts including the neurodevelopmental disorders discussed above. By contrast, derivatives of Roscovitine, such as those pioneered by the Meriney group, are logical candidates for further development (Tarr et al., 2013; Wu et al., 2018). Another possibility, which may not be a stretch given nascent cryo-EM images and the increasingly frequent implementation of deep learning approaches, is the modification of the L-type channel agonist (-)Bay K 8644 for use as a specific P/Q-type channel agonist (Zhao et al., 2019).

Despite these advantages, the zebrafish model system does pose some challenges. The fact that gene duplication endowed teleosts with two* cacna1a* genes can be problematic, even though the characterization of the *tb204a* mutant revealed that *cacna1aa* channel isoform makes little, if any, contribution to neurotransmission at the NMJ (Wen et al., 2013). However, sequence similarity between the isoforms may complicate knockdown experiments using antisense strategies and the production of reliable antibodies. Finally, zebrafish, like flies and mice, are not human. Nonetheless, the flexibility of the fish model makes it potentially useful as a first-line indicator of individual mutations and a vehicle for the development of personalized therapies.

## Conclusions

Whole-exome sequencing is bringing new Ca_V_2.1 mutations out of the woodwork (see Damaj et al., 2015; Jiang et al., 2016; Weyhrauch et al., 2016; Luo et al., 2017; Travaglini et al., 2017). Many of the syndromes caused by these point mutations are more severe than the typical EA2 and FHM1 in that they present with not only ataxia or migraine but also with neurodevelopmental delay, nystagmus, epilepsy, cerebellar degeneration, hypotonia, and cognitive dysfunction. Modeling these more severe disorders is problematic because of the heterogeneous effects on channel function and the limitations intrinsic to flies and mice. Although not without some disadvantages, zebrafish present a useful model system for the timely characterization of pathological phenotypes and pharmacological correction.

## Author Contributions

ST, AR, and RB wrote the article. All authors read and approved the final manuscript.

## Conflict of Interest

The authors declare that the research was conducted in the absence of any commercial or financial relationships that could be construed as a potential conflict of interest.
